# Motor Learning Characterized by Changing Lévy Distributions

**DOI:** 10.1371/journal.pone.0005998

**Published:** 2009-06-22

**Authors:** Tyler Cluff, Ramesh Balasubramaniam

**Affiliations:** 1 Sensorimotor Neuroscience Laboratory, Department of Kinesiology, McMaster University, Hamilton, Ontario, Canada; 2 McMaster Integrative Neuroscience Discovery and Study (MiNDS), McMaster University, Hamilton, Ontario, Canada; Mount Sinai School of Medicine, United States of America

## Abstract

The probability distributions for changes in transverse plane fingertip speed are Lévy distributed in human pole balancing. Six subjects learned to balance a pole on their index finger over three sessions while sitting and standing. The Lévy or decay exponent decreased as a function of learning, showing reduced decay in the probability for large speed steps and was significantly smaller in the sitting condition. However, the probability distribution for changes in fingertip speed was truncated so that the probability for large steps was reduced in this condition. These results show a learning-induced tolerance for large speed step sizes and demonstrate that motor learning in continuous tasks may be characterized by changing distributions that reflect sensorimotor skill acquisition.

## Introduction

Traditionally, motor learning for continuous, goal-directed movements has been difficult to characterize. Although dynamical approaches argue motor learning results from the evolution of potential landscapes [1], what has been elusive is how to quantify and interpret the change behaviourally. On the other hand, motor learning for discrete movements can be classified on the basis of performance error, for example in end-effector position relative to some salient target [2]. These studies have a long-standing history in the literature and consequently, have revealed much more about motor learning for discrete tasks [3]–[6].

More recently, studies that examined learning for continuous tasks have incorporated a technique from control systems theory – the Kalman filter. The Kalman filter is a linear estimator that in neural applications recursively determines the sensory consequences of movement based on the intended motor command [7]–[9]. The Kalman filter is optimal for linear systems because it minimizes least-square estimation error [10], [11]. Commonly used computational approaches in motor control argue the Central Nervous System (CNS) employs internal forward models in tasks such as visuomotor pole balancing. Predictive control can be favorable for the neural control of pole balancing because it can help circumvent sensorimotor processing delays to produce low latency movements required for maintaining pole stability [12]. Predictive mechanisms enable perturbations/threats to stability to be anticipated and accordingly, corrections can be performed in anticipation of these disturbances. More generally, predictive mechanisms are thought to be implicated in minimizing body-state estimate uncertainty; the continuous evolution of limb position in response to motor inputs [12]–[15].

Contrary to the abovementioned mechanisms, pole dynamics may be stabilized by stochastic properties characteristic of motor control [16]–[18]. The findings of Cabrera and Milton demonstrate that fingertip speed profiles in human pole balancing show power law scaling. Power law scaling was also evident in the laminar phases (time intervals) for successive corrective movements, which demonstrated that corrective movements were intermittent in human pole balancing. In confirmation of intermittent control, behavioral data demonstrated that 98% of corrective movements were shorter than our sensory processing delays. Numerical analyses have since demonstrated that balance is facilitated in time-delayed stochastic systems, provided the system is tuned near a stability boundary. In this case, control could result from stochastic processes that force the fingertip trajectory back and forth across stability boundaries [17], [18]. On the basis of efficiency, intermittent or ‘bang-bang’ control might be favored to continuous estimation in stochastic, time-delayed systems since the computational burden incurred by the CNS is minimized [19].

Systems characterized by on-off intermittency exhibit two distinct phenomenological states. In the “off” state, dynamic variables remain approximately constant over various time intervals. Conversely, the “on” state is characterized by intermittent bursting of the dynamical variable. Intermittency requires the underlying system to possess an invariant subspace, whereby provided the dynamical variable remains within the bounds of the invariant space, it remains relatively quiescent. This bound is manifest as a threshold, that when crossed, results in subsequent bursting of the dynamical variable, ie., the system transitions from the “off” to “on” state [20].

In this context, the stochastic process that characterizes fingertip speed profiles is given by a symmetric Lévy process *L_α_* (Δ*s*, Δ*t*) given by [17]: 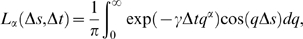 where Δ*s* is the fingertip speed step size, Δ*t* is the interval between successive observations, γ is the scaling factor (*γ*>0) and *α* is the Lévy index (0<*α*<2). The Lévy process is an unbounded, unconstrained random walker. The unbounded, asymptotic character of the Lévy distribution results in an infinitely variant process, resulting in the absence of the first and second statistical moments. It is characterized by both ‘slow’ and ‘fast’ components, what are referred to as ‘rambling’ and ‘trembling’ in posture research, respectively [21], [22]. As mentioned, the slow and fast regimes of the Lévy process are demarcated by a critical threshold; when the dynamic variable of consideration is within the confines of this threshold the process is in the slow (small amplitude fluctuations) regime and consequently, is free to vary.

Previously, research demonstrated the probability for large fingertip speed steps increased with learning in a human pole balancing task [17], which the authors argued was indicative of tolerance to stochastic processes. This consideration has important ramifications for our understanding of motor learning. In the event that the distribution broadens with learning, this corresponds to a smaller decay in the probability for large step sizes. Behaviorally, this is manifest as tolerance to stochastic processes: the participant becomes more tolerant to large changes in fingertip speed as proficiency in the pole balancing task increases. The purposes of this study were two-fold: first, to determine whether the decay exponent for the probability of a given step size, *α*, changed with learning, and second, to determine whether *α* varied in a sitting versus standing condition. We include the sit versus stand contrast to highlight differences in control between two conditions that differ markedly in the available biomechanical degrees of freedom. We expected that the probability for large changes in fingertip speed would be increased in the standing condition.

## Methods

Six healthy subjects (2 male, aged 26–28 years; 4 female, aged 23–27 years) participated in this research. The sample was a convenience sample (subjects were members of the Sensorimotor Neuroscience Laboratory). Procedures were performed in accordance with the Declaration of Helsinki with subjects providing written informed consent prior to experimentation. The protocol was approved by the University of Ottawa institutional review board. Subjects balanced a wooden dowel with length 62 cm, diameter 0.635 cm and mass 50 g in two experimental conditions: sitting and standing. Sitting trials were performed with subjects seated comfortably in a chair at the subjects' preferred seat height. Subjects were required to perform pole balancing with their back remaining in contact with the seat. In the standing condition, subjects performed pole balancing with feet approximately shoulder-width apart. Subjects were free to move the upper body. However, subjects were not allowed to move their feet in the standing condition. In the event that foot movement occurred, these trials were discarded and excluded from subsequent analyses.

Motion capture was performed with 8 VICON MX-40+ infrared cameras sampled at 500 Hz (Denver, CO, USA). We tracked pole motion in three-dimensions using two spherical reflective markers (14 mm diameter) affixed to the top and bottom of the pole with double-sided adhesive. Marker trajectories were processed offline with the Workstation software and exported to MATLAB™ (Mathworks, Natick, MA) for subsequent analysis.

This study employed a learning protocol: subjects learned pole balancing over a two week period. Data collection occurred on the first day, followed by subsequent collection every fourth day. Confounded learning effects that may have resulted from the ordering of conditions were avoided by counterbalancing the order of conditions across subjects. On days where data was not collected subjects performed 30 min of practice (15 min per condition), distributed according to their preference.

The Lévy process (*α*<1.2) requires 10^5^–10^6^ samples to be distinguished reliably from the Gaussian process (0<*α*≤2). With our sampling rate (500 Hz), this corresponded to a minimum balancing time of 200 s. Individual trials for each condition and session were parsed into a single aggregate trial for each subject. All data presented here were derived from de-trended, aggregate fingertip speed profiles > than 5×10^5^ samples (1000 s). We collated time-series data of the changes in fingertip speed (Δ*s*). We believe this is the more effective means of collating trials because it minimizes artefact that might result from introducing particular speed-steps- the effect of parsing would be accentuated by making the aggregate trial with fingertip positions and two-point differentiating to determine fingertip speed. Moreover, the effect of parsing individual trials to form a single aggregate trial would be minimized by the number of data points in relation to the number of trials parsed to form the aggregate time-series (max 50 trials vs. >500000 samples per aggregate time-series). Similar to Cabrera and Milton [17], we examined the corrective movements that occurred on time scales shorter than or to the same order as the neural delay.

We computed the 2-D fingertip speed, 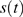: 
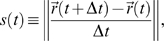
 where 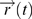 corresponded to the transverse plane 2-D fingertip position at time *t*; 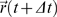 was the fingertip position at time *t*+Δ*t*; and Δ*t* represented the time step between successive observations. 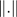 represented the vector norm. Therefore, 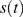 was the Euclidean fingertip speed. The de-trended speed 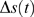 was computed as: 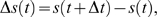 where 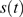 was the 2-D velocity norm at time *t* and 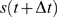 was fingertip speed at *t*+Δ*t*. It should be noted this expression corresponds to the de-trended speed and not acceleration. This expression removes any time dependent linear trend and is therefore equivalent to the high-pass filtered speed [23].

We computed the probability of a given step size, *P*(Δ*s*, Δ*t*) by plotting histograms with bin size set to 1 mm/s. To determine whether the probability of a given step size was influenced by the time between observations, Δ*t*, we decimated Δ*s*(*t*) on a logarithmic scale by factors 1 to 1000. We plotted the probability of return (i.e., the probability of zero change in fingertip speed between observations), *P*(0, Δ*t*), as a function of Δ*t*. The power law exponent *α* was computed from the relationship 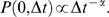 In other words, *α* was computed by regressing *P*(0, Δ*t*) onto Δ*t* on a log-log scale. We contrasted the power law exponent (*α*) across sessions (3) and conditions (2) using a 3×2 ANOVA with repeated-measures. Post-hoc analysis was performed with Bonferonni corrections. The significance level for statistical contrasts was 0.05. As a measure of performance we quantified mean balancing time and contrasted the dependent measure across sessions (3) and conditions (2) using a 3×2 ANOVA with repeated-measures. The balancing time *t_bal_* (in seconds) for individual trials was determined from the number of samples as 
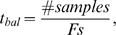
 where *Fs* was the sampling frequency for data collection. Mean balancing time 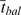 was defined as the arithmetic average of the time spent pole balancing across trials for each session and condition, 
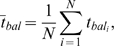
 where *N* was the number of trials and *τ* was the balancing time (in seconds) for individual trials, respectively. Post-hoc analysis was performed with Bonferonni corrections.

## Results

**Figure 1:**
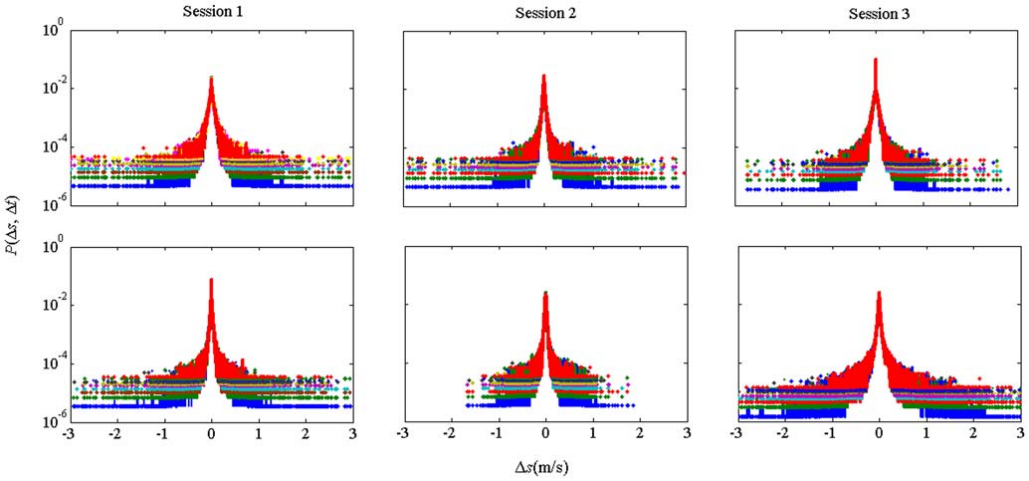
Change in speed is Lévy distributed in the visuomotor stick-balancing task. Decimated time-series shows the probability of a given step size *P*(Δ*v*, Δ*t*) is influenced by the time between successive observations, Δ*t* (0.002 to 2 s). Overlaid colors represent the decimated time-series, with time-steps ranging from 0.002 to 2 s (Δ*t* (0.002 to 2 s). *a*) Sitting; *b*) Standing condition. Left to right: Sessions 1 to 3.

**Figure 2:**
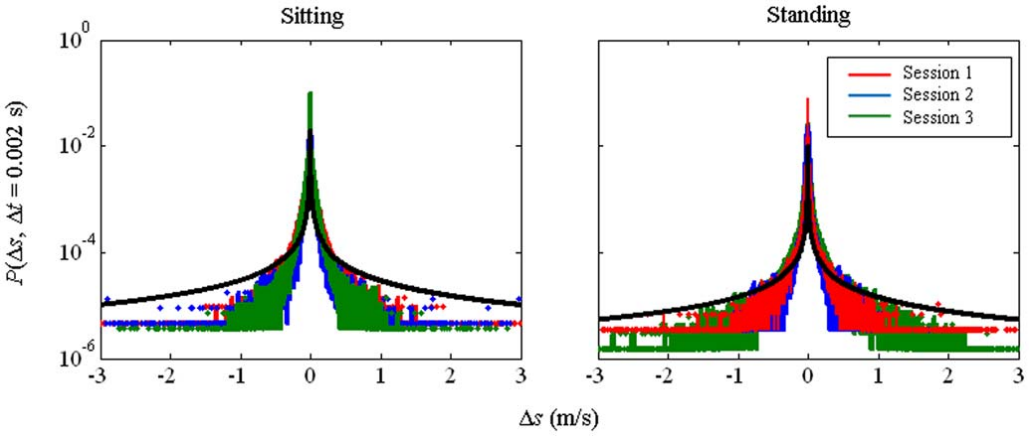
*P*(Δ*s*, Δ*t* = 0.002 s) over three sessions in the sitting (left) and standing (right) conditions. Red: Session 1; Blue: Session 2; Green: Session 3. Solid black line represents theoretical Lévy distribution with a) *α* = 0.95 and scale parameter *γ = *0.03, b) *α* = 0.98 and scale parameter *γ* = 0.025. The overlaid theoretical Lévy distribution demonstrates both decay exponent *α* and truncation change with learning in the standing condition.

Figure 1 shows representative data for the probability of a given step size *P*(Δ*s*, Δ*t*) in sitting (top row) and standing conditions (bottom row), as well as across sessions (1: left; 2: middle; 3: right column) with decimated time-series. Figure 2 shows representative data for Subject 1 in sitting and standing conditions for the three experimental sessions (Session 1: Red; Session 2: Blue; Session 3: Green). Both figures demonstrate the distribution broadened with learning, rendering the probability of large step sizes *P*(Δ*s*, Δ*t*) significantly greater with experience. Figure 2 also shows theoretical Lévy distributions with parameters determined from Session 1 data (solid black line). For the sitting condition, the fit parameters were decay exponent *α* = 0.95 and scale parameter *γ* = 0.03, whereas for the standing condition the parameters were *α* = 0.98 and *γ* = 0.025. As shown, the central region of the experimental distributions for all three sessions are reasonably well fit by the parameters determined from session 1, which suggests that both decay exponents and truncation are influenced by learning.

**Figure 3:**
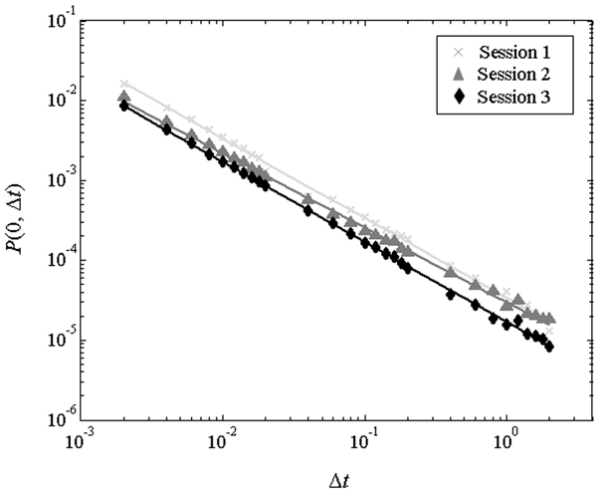
*P*(0, Δ*t*) follows a power-law distribution for Δ*t* = 0.002 to 2 s, in the sitting condition.

We decimated the change in velocity time-series Δ*s*(*t*) to determine the probability of return *P*(0, Δ*t*) or zero-speed when the time between observations was varied between 0.002 to 2 s. The decay exponent *α* was estimated by regressing *P*(0, Δ*t*) onto Δ*t* on double-log plot, depicted in Figure 3. The decay exponent *α* was computed for each session and condition. Statistical analyses demonstrated *α* was dependent on both session, *F* (2, 10) = 7.889, *P* = 0.009, and condition, *F* (1, 5) = 7.696, *P* = 0.039 where *F* represents the Fisher statistic for the contrast (mean-square error within-subjects/mean-squared error between-subjects), (2, 10) represents the statistical degrees of freedom for the mean-squared error within- and between-subjects, respectively. In this context, *P* represents the probability of observing the same or more extreme results. With respect to the session effect, *α* was reduced in session 3 (*M* = 0.935, *SE* = 0.009) relative to session 1 (*M* = 0.981, *SE* = 0.011), *P* = 0.046 (Figure S1). The decay exponent, *α* was similar between session 2 (*M* = 0.964, *SE* = 0.014) and sessions 1 and 3 (*P*>0.05). Lastly, the decay exponent *α* was significantly larger in the standing (*M* = 0.973, *SE* = 0.011) relative to sitting (*M* = 0.947, *SE* = 0.009) condition, *P* = 0.039 (Figure S2). The decay exponent *α* was not influenced by a condition × session interaction effect (*F* (2, 10) = 0.942, *P*>0.05).

Balancing time was the average time spent pole balancing for each session and condition and was contrasted via a 3 (session) ×2 (condition) ANOVA with repeated-measures. Balancing time was dependent on both session, *F* (2, 10) = 14.331, *P* = 0.001, and condition, *F* (1, 5) = 6.919, *P* = 0.047, but was not influenced by a session × condition interaction, *F* (2, 10) = 2.916, *P*>0.05. Regarding the session effect, balancing was greater in session 3 (*M* = 73.686, *SE* = 12.7239) relative to session 1 (*M* = 29.796, *SE = *11.782) (*P* = 0.034) and session 2 (*M* = 44.503, *SE* = 11.8211) *P* = 0.034, whereas mean balancing times for sessions 1 and 2 were significantly different from one another, *P*>0.05 (Figure S3). Lastly, mean balancing time was significantly greater in the standing (*M* = 59.613, *SE = *14.104) relative to sitting condition (*M* = 39.0436, *SE* = 11.0446), *P* = 0.047 (Figure S4).

## Discussion

The key goal of our study was to determine how power law scaling in human pole balancing was influenced by learning. Previously, Cabrera and Milton demonstrated that learning resulted in less severely truncated distributions for the probability of large step velocities, *P*(Δ*s*, Δ*t*). The authors proposed the change resulted from truncation. With truncation, the symmetric Lévy distribution becomes 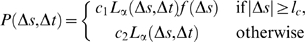
where *P*(Δ*s*, Δ*t*) is the probability of a given velocity step, *c_1_*and *c_2_* are normalization constants, *L_α_*(Δ*s*, Δ*t*) is the symmetric Lévy distribution, *f*(Δ*s*) is the truncation function, Δ*s* is the step-size and *l_c_* is the threshold for truncation. The truncation function *f*(Δ*s*), can be approximated as [24], 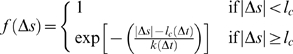 where Δ*s* is the change in velocity, Δ*t* is the time-step, *l*_c_ is the truncation threshold and *β* is (*α*−2). In theory, the independent axis of the Lévy distribution spans infinitely and therefore does not have first and second statistical moments [23]. Cabrera and Milton proposed increased probability for large changes in fingertip speed (Δ*s*) resulted from changes in truncation and not scaling. We were concerned with whether the observed changes in the probability distribution for step size resulted not only from truncation, but also from a reduction in the decay exponent *α* for *P*(Δ*s*, Δ*t*). Our hypothesis was confirmed in that power law scaling was influenced by learning: participants became more tolerant of large changes in fingertip speed and this was reflected in the decay exponent, *α*.

Moreover, we contrasted decay exponents (*α*) for *P*(Δ*s*, Δ*t*) in sitting and standing conditions. Our results suggest that while decay exponents for the probability of a given step size (Δ*s*) were significantly larger in the standing versus sitting condition, the *P*(Δ*s*, Δ*t*) distribution was considerably wider when standing. Therefore, individuals were relatively more tolerant of large fingertip excursions when standing. At first pass, these results seem counterintuitive. The decay exponent for *P*(Δ*s*, Δ*t*) was reduced when sitting relative to standing, indicating the decay in probability for large changes in fingertip speed was less severe. We argue, in confirmation of the results suggested by Cabrera and Milton [17] that truncation was more severe in the sitting condition – the physical capacities of the system were exhausted – and individuals were not capable of tolerating large step sizes to the same extent. The hypothesis is further supported by Figure 2, which demonstrated the experimental distributions for all three sessions were reasonably well fit in the central region by a theoretical distribution plotted using parameters *α* and *γ* determined from session 1 data, for both sitting and standing conditions. These results follow a more generalized formulation of truncation. With respect to the discontinuous truncation function *f*(Δ*s*) mentioned above, there are three broad truncation classifications: (i) the distribution is truncated gradually from return [25], *l_c_* = 0; (ii) the distribution deviates from the symmetric Lévy and is truncated gradually from some critical change in velocity, *l_c_ ≠* 0; and (iii) the truncation gain is zero, *k* = 0, and system capacities are exhausted rapidly at the critical step size [26], *l*_c_. Physically, the truncation may have resulted from a reduction in degrees of freedom, ultimately reducing the range of motion and consequently, the truncation gain *k*. Currently, we are working to identify the specific truncation mechanisms computationally.

Conceivably, changes in the distribution of fingertip speed changes *P*(Δ*s*, Δ*t*) could have occurred in the absence of learning. However, we quantified a performance measure for pole balancing based on balancing time. Balancing time was the mean time spent pole balancing for each session and condition. We found that learning did occur; the time spent balancing was dependent on a session effect, which demonstrated that performance improved with learning. Regarding the classification of participant skill levels, it is likely that participants were still of low-moderate level since mean balancing times were less than one minute [27]. Future research should consider the differences in the examined distributions between low-moderate and expert pole balancers (mean balancing time ≫1 minute). As demonstrated, changes in the Lévy index certainly occur for the progression from low to moderate skill; it is unlikely these changes persist with further developments of expertise.

Our sit versus stand comparison was conducted to delineate the mechanisms by which individuals learn to accommodate noise in pole balancing. Power law scaling is known to arise in unstable physical systems influenced by parametric noise [16], [17], [28], [29]. Balance control in unstable, time-delayed dynamical systems can benefit from the presence of parametric noise, provided the system is placed near a stability boundary. In such a way, the unstable inverted pendulum with time-delayed (somatosensory) feedback is stabilized by parametric noise- stochastic forcing of a gain term back and forth across a stability boundary [28]. The rationale for the sit vs. stand comparison can be summarized as follows. Sensorimotor noise is state-dependent [30]. The standing condition employed here capitalizes on more biomechanical degrees of freedom relative to the sitting condition, with state-dependent noise inherent to each additional degree of freedom. In alignment with the premise that balance control can benefit from noise, we hypothesized that pole balancing would be facilitated in the standing condition. The hypothesis was confirmed, since greater contribution from the distribution tails were observed in the standing condition and participants were capable of balancing for a prolonged period relative to when sitting. Though at present we know little about the underlying mechanism, one explanation might be that abundant dimensions along which the system can vary (muscle activations, joint kinematics) facilitate the pole balancing task.

Previously, research into the mechanisms underlying postural control demonstrated that performance in a dual task (counting backwards by 3′s) with eyes closed reduced postural fluctuations relative to an eyes closed condition. Similarly pole balancing performance improved for a moderately skilled subject in a dual-task situation (improvements were observed for both rhythmical leg movement and imaged movement) [cf. [31] for exemplary video, [27] for more detail]. In short, these studies suggest that maintaining balance might be an exception for motor performance, since in general; dual task conditions have a deleterious effect on performance. Balance might be the exception since it appears to be facilitated by passive dynamics of the neuromuscular system (ligaments, joint capsules). However, there are active contributions to the control of balancing that are both reflexive (muscle spindles, golgi tendon organs) and voluntary contributions (muscle contraction and tension at the tendon insertion) [32]. In the present research we make no distinction between learning in balancing studies or other conventional motor learning studies (finger tapping intervals, bimanual coordination). We speak of learning strictly in the sense that performance improved as a function of practice, which is synonymous to learning for many tasks in the motor domain.

In summary, we demonstrated that motor learning resulted in increased tolerance for large pole displacements in a human pole balancing task. The decay exponent *α* was influenced by learning, becoming significantly smaller with experience and resulting in less severe decay in the probability for a given velocity step size, *P*(Δ*s*, Δ*t*). Moreover, the decay exponent *α* for *P*(Δ*s*, Δ*t*) was greater in a sitting versus standing condition. Our results show conspicuously that both decay exponents and truncation change with learning, resulting in an increased tolerance to large fingertip excursions in pole balancing.

Previously, Cabrera and Milton [16] demonstrated that time intervals between corrective movements followed a −3/2 power law. These results were argued to be indicative of intermittent control. Cabrera and Milton [18] argue that intermittent control is favorable to continuous estimation on the basis of efficiency - an intermittent control strategy would moderate the computational burden incurred by the CNS. In their view, pole balancing dynamics and the corresponding intermittent control regime usurps any need for continuous estimation by the CNS since passive or noisy pole dynamics act to impart a dynamical stability. The CNS need only enact control when pole dynamics cross a stability boundary, represented as threshold [16]–[18]. Such dynamics have previously been reported in the control of upright posture [33]. When the threshold is surpassed, the system transitions from the fast to slow regime, defined by translations of the pole pivot.

In this model, the ability to sense threshold crossings for pole dynamics is bounded by the limitations of sensory processing. Sensory feedback involves processing delay, which incorporates limitations in transduction, conduction velocity, multi-modal sensory integration, and neural processing to enable a control decision. When a movement decision is made, motor commands descend from the primary motor cortex to the distal effectors. Continuous estimation (predictive) can help circumvent sensory processing delay. That is, pole excursions might be represented probabilistically in terms of velocity steps and the probability of these steps occurring. This proposal is aligned with functional imaging studies that support a modular architecture in the cerebellum for internal object representations [34].

In a recent experiment, manipulating an object with complex dynamics (subjects balanced a flexible weighted ruler by applying a force to the tip) resulted in greater activation of the ipsilateral anterior cerebellum relative to an object with simple dynamics [35]. Activation of the ipsilateral anterior cerebellum was similar to that observed in a previous study [36] and was attributed to the acquisition of an internal representation of the task. The prevailing question then is why the cerebellum shows differential activation when controlling objects with complex relative to simple dynamics. These data imply that activation of the cerebellum is modulated by task complexity. In the pole balancing task the observed corrections are intermittent as demonstrated by a −3/2 power law for time intervals between corrective movements [16]; it appears that the motion of the pole is corrected only when a fall is impending. It could be that the underlying control mechanism is continuous and predictive: with learning a representation of pole dynamics might be built and consequently used to estimate finger and pole states so as to circumvent neural processing delays.

Predictive control mechanisms can help circumvent neural processing delays by anticipating perturbations and performing corrective movements prior to or as these perturbations arise. However, whether an estimation based model can replicate the intermittency observed in our experimental data is not yet known. Though an estimation strategy might represent a plausible control mechanism for the CNS, other scenarios might include a mixture of non-predictive and predictive mechanisms. In this context, participants might rely on passive dynamics until a stability threshold is surpassed, at which point a predictive strategy might be enacted for correction.

The present finding that the joint probability distribution for changes in fingertip speed over time intervals is Lévy distributed challenges any mechanism based solely on prediction. Typically, Lévy distributed processes are thought to be reflective of non-predictive searches or foraging patterns [37], [38], which is problematic for a theory of predictive control for pole balancing. As a further consideration, the current understanding of predictive mechanisms in motor control is grounded in Kalman filter-based models. The limitation of the conventional Kalman filter as applied to the context of the current results is that it implicitly assumes additive Gaussian process and measurement noise [39] and not the multiplicative noise that typically gives rise to power-law distributed variables. These considerations are not easily explained by current predictive theories of motor control. Future endeavours should take these findings into consideration since important insights into the mechanisms governing the control of unstable systems may be proffered, including the possibility that the interplay between passive and predictive mechanisms (intermittency) might change as a function of expertise. Similarly, future studies will likely incorporate functional imaging to determine whether activation of the cerebellum while performing a virtual pole balancing task is dependent on learning, both in terms of intensity and the functional loci of activity. Though previous research suggests overlearning beyond asymptotic performance is reflected in increased cerebellar activation with expertise in a complex bimanual coordination task [40], it is not known whether similar effects can be expected in balancing tasks.

## Supporting Information

Figure S1 The decay exponent *α* was greater in the standing relative to sitting condition, signifying more stringent decay in the probability for large step sizes in the standing relative to sitting condition.
(0.05 MB TIF)

Figure S2 The decay exponent *α* was dependent on learning, resulting in less stringent decay in the probability for large step sizes in the third relative to first session.
(0.05 MB TIF)

Figure S3 Mean balancing time tbal was dependent on condition, with time spent balancing significantly greater in the standing relative to sitting condition.
(0.05 MB TIF)

Figure S4 Mean balancing time t_bal increased with learning, with time spent balancing significantly greater in the third relative to first session.
(0.04 MB TIF)
