# Supplementary figures and images for: Motor Learning Characterized by Changing Lévy Distributions
Source: PLoS One. 2009 Jun 22;4(6):e5998. doi: 10.1371/journal.pone.0005998 (PMC2695787; doi:10.1371/journal.pone.0005998)

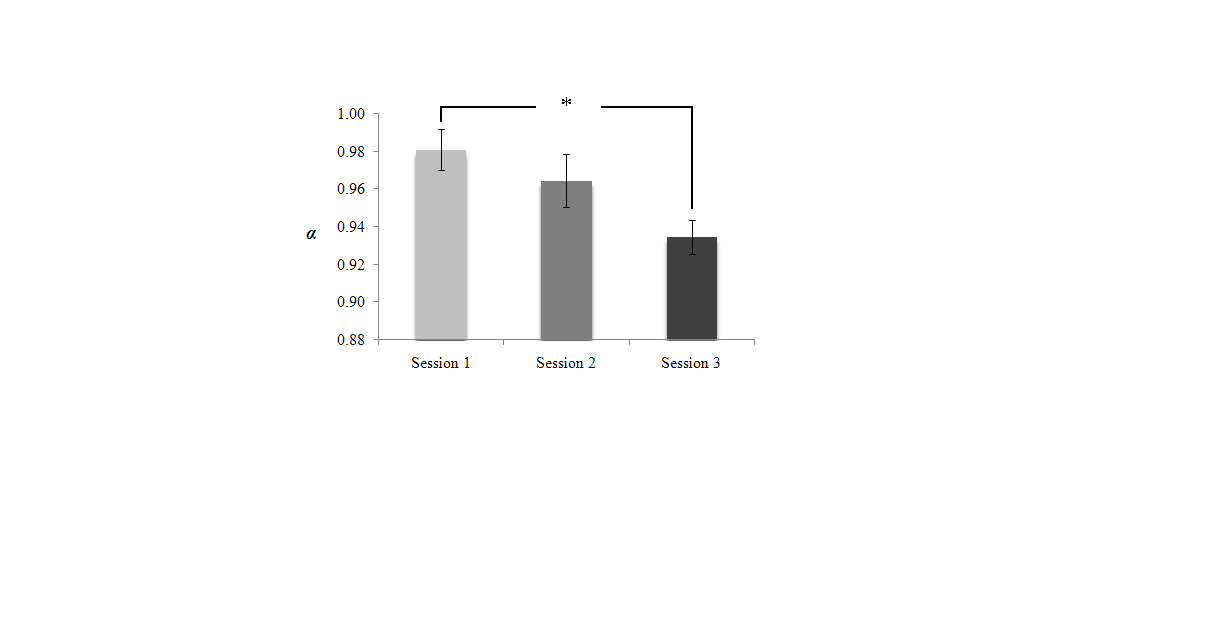

Supplement: Figure S1 — The decay exponent α was greater in the standing relative to sitting condition, signifying more stringent decay in the probability for large step sizes in the standing relative to sitting condition. (0.05 MB TIF) [file pone.0005998.s001.tif]

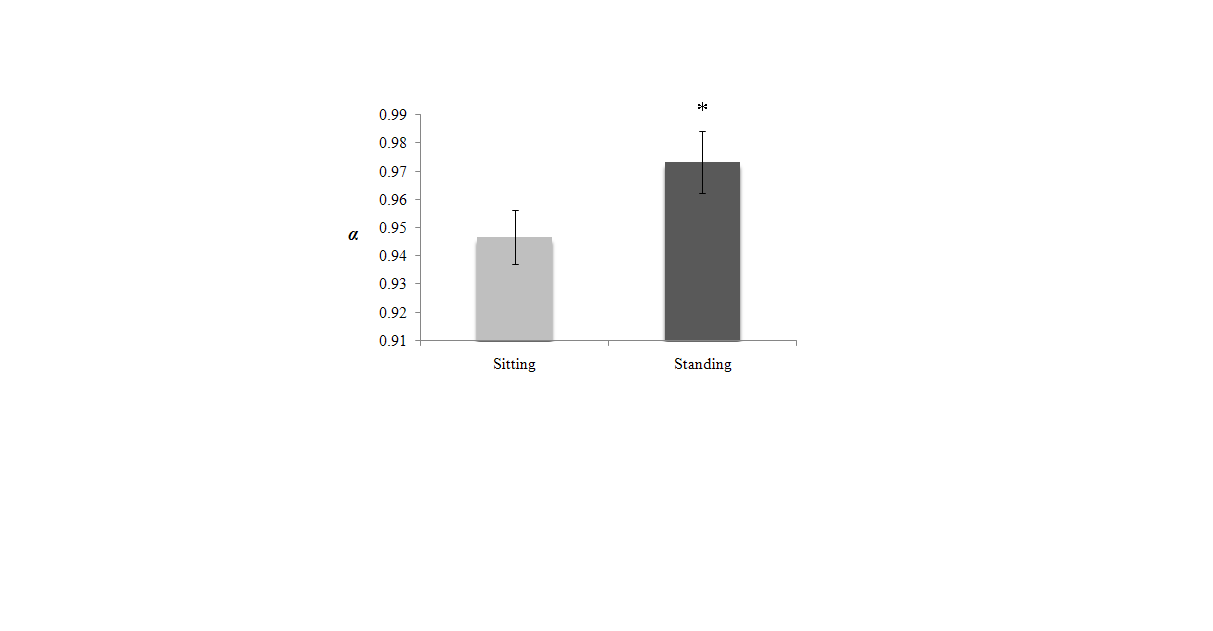

Supplement: Figure S2 — The decay exponent α was dependent on learning, resulting in less stringent decay in the probability for large step sizes in the third relative to first session. (0.05 MB TIF) [file pone.0005998.s002.tif]

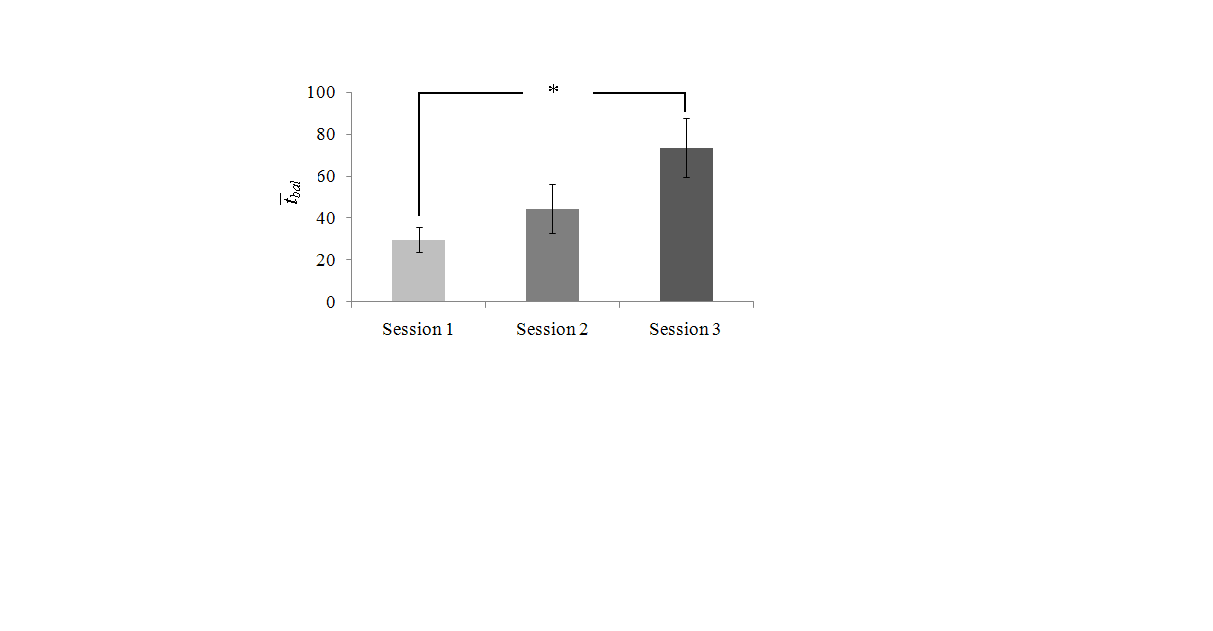

Supplement: Figure S3 — Mean balancing time tbal was dependent on condition, with time spent balancing significantly greater in the standing relative to sitting condition. (0.05 MB TIF) [file pone.0005998.s003.tif]

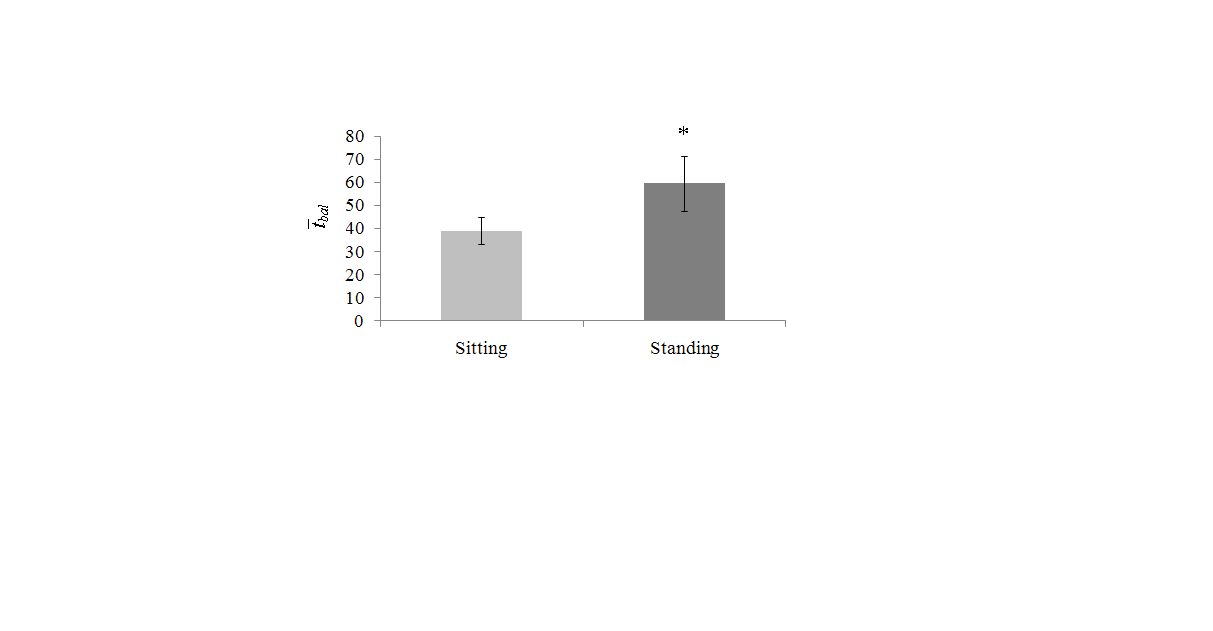

Supplement: Figure S4 — Mean balancing time t_bal increased with learning, with time spent balancing significantly greater in the third relative to first session. (0.04 MB TIF) [file pone.0005998.s004.tif]
